# The poverty-related neglected diseases: Why basic research matters

**DOI:** 10.1371/journal.pbio.2004186

**Published:** 2017-11-09

**Authors:** Peter J. Hotez

**Affiliations:** 1 Texas Children’s Hospital Center for Vaccine Development, National School of Tropical Medicine, Baylor College of Medicine, Houston, Texas, United States of America; 2 Department of Biology, Baylor University, Waco, Texas, United States of America

## Abstract

Together, malaria and the neglected tropical diseases (NTDs) kill more than 800,000 people annually, while creating long-term disability in millions more. International support for mass drug administration, bed nets, and other preventive measures has resulted in huge public health gains, while support for translational research is leading to the development of some new neglected disease drugs, diagnostics, and vaccines. However, funding for basic science research has not kept up, such that we are missing opportunities to create a more innovative pipeline of control tools for parasitic and related diseases. There is an urgent need to expand basic science approaches for neglected diseases, especially in the areas of systems biology and immunology; ecology, evolution, and mathematical biology; functional and comparative OMICs; gene editing; expanded use of model organisms; and a new single-cell combinatorial indexing RNA sequencing approach. The world’s poor deserve access to innovation for neglected diseases. It should be considered a fundamental human right.

The latest estimates from the Global Burden of Disease Study 2015 show an astonishing public health impact from malaria and the neglected tropical diseases (NTDs), the latter referring to a group of poverty-promoting and debilitating parasitic and related infectious diseases. The estimates indicate that together, malaria and the NTDs caused 843,100 deaths in 2015 [1] and 79 million disability-adjusted life years (DALYs) [2], such that these conditions rank near the top of all global health threats.

Beginning in 2000 and under the auspices of a set of Millennium Development Goals, the major industrialized countries, led by the United States, United Kingdom, and European Union, provided large-scale support for malaria control through the US President’s Malaria Initiative and a Global Fund to Fight AIDS, Tuberculosis, and Malaria, in addition to packages of essential NTD medicines for a group of 6 parasitic infections and trachoma [3]. According to the Kaiser Family Foundation, the US government alone now spends around $8 billion annually for large-scale global control programs, which includes those for malaria and NTDs, as well as HIV/AIDS, tuberculosis, and maternal and child health [4].

International funding was also increasingly directed to support global health research, a major piece of which funds translational medicine and programs to promote the research & development (R&D) for new drugs, diagnostics, vaccines, and vector control approaches. Such translational R&D initiatives include funding from the US government through the National Institute of Allergy and Infectious Diseases (NIAID) of the National Institutes of Health (NIH), as well as important private foundations such as the Bill & Melinda Gates Foundation and the Wellcome Trust.

The years between 2000 and 2010 are sometimes referred to as the “decade of global health,” as the donor and international communities committed unprecedented funds for large-scale public health interventions and began supporting nonprofit product development partnerships (PDPs) to begin the arduous process of developing new interventions. At the start of the global health decade, I launched a new basic science department that emphasized fundamental research on pathogen–host interactions for poverty-related neglected diseases. One of my striking observations then was the extreme difficulties that my junior faculty members faced obtaining their first grant from the NIH and other sources. Unfortunately, this situation still continues today as we pass through our second decade of flat-line NIH funding.

In my newer role (as an academic dean), I have become increasingly worried that by shortchanging fundamental and basic research on tropical disease pathogens and host–pathogen interactions we are exhausting new ideas and approaches for achieving new cures and preventive measures.

Indeed, a recent analysis of new products for neglected diseases indicates that we urgently need a more robust pipeline [5], which in turn would benefit from greater innovation through basic science research. Today, we may not have a sufficient understanding of basic life science processes to generate enough novel drugs, diagnostics, and vaccines.

Unfortunately, global funding for basic research is not adequate. According to the most recent G-FINDER Report (issued annually to report on global investments in neglected disease R&D) that assesses the finances of neglected diseases R&D, it is estimated that of the approximately $1.5 billion spent globally on R&D for malaria and NTDs in 2015, only about one quarter went to support basic research [6]. This amount represents a modest global investment and is an indication that basic research for neglected disease pathogens is undervalued.

The bottom line is that in order to develop new technologies to benefit the world’s poorest people, we’ll need to focus more resources on some key areas of basic research. Without co-investments in basic research, it is unlikely we’ll see many game-changing neglected disease biotechnologies anytime soon.

There are several major areas of basic and fundamental research that urgently require investments. Highlighted here are some key initiatives that deserve our attention, which, if better supported, could accelerate new innovations for the poverty-related neglected diseases (Box 1). The areas highlighted here are not one-off activities. Instead, they were selected based on their overlapping missions and ability to reinforce each other, with an emphasis on new molecular and cellular processes and how they integrate into systems and disease ecology.

**Box 1. Key areas of basic and fundamental research to benefit the world’s poor.**

- Whole organism biology and life histories
- Model organisms and single-cell combinatorial indexing RNA sequencing (sci-RNA-seq)
- Functional and comparative OMICs and gene editing
- Ecology, evolution, and mathematical biology
- Systems biology and immunology

## Whole organism biology and life histories

There is still much to learn about our basic concepts, life history stages, and even life cycles of medically important parasites. Much of our knowledge in this area was generated in the early part of the 20th century but was never revisited using modern approaches. For example, the life cycle of *Strongyloides stercoralis* was established by Friedrich Fuelleborn during the 1910s and later in the 1920s by Masao Nishigoii, who found evidence for autoinfection, but then was not systematically reinvestigated for 70 years until Gerhard Schad and Linda Mansfield used modern radiotracing methods to reveal multiple alternative pathways by which infective larvae migrate through body tissues to become adult worms [7, 8]. Similarly, R. A. Wilson and his colleagues have identified novel mammalian tissue migration pathways for schistosomes [9]. It’s conceivable that similar findings could be made for many other human parasites. Today, graduate and postdoctoral training is heavily focused on molecular and cellular studies, but often at the expense of whole organism biology. It is true that for neglected disease pathogens, the field of molecular parasitology that took off beginning in the 1980s has yielded important and fundamental insights as well as innovative approaches to drugs, diagnostics, and vaccine development [10]. But our collective expertise in the biology of whole organisms may be disappearing with recent retirements, and, so far, there have been minimal efforts to develop restorative training programs in this area.

## Model organisms and single-cell combinatorial indexing RNA sequencing

Many of our greatest scientific discoveries in molecular biology were achieved using model organisms such as *Caenorhabditis elegans* and *Drosophila melanogaster*. We have started to make progress in tapping into the rich diversity of technologies developed for such model systems and applying them towards neglected pathogens [11–13], but this approach is still underutilized. In a new development, the genomics group at the University of Washington published a single-cell combinatorial indexing RNA sequencing (sci-RNA-seq) approach to allow for a molecular cell-by-cell profile of *C*. *elegans* [14]. Similar approaches could identify rare cell types in parasitic helminths or cell types unique to parasites, which could serve as the basis for new therapeutic approaches.

## Functional and comparative OMICs and gene editing

In related studies, a central repository of genes from *C*. *elegans* and other free-living and parasitic nematodes has been curated into a WormBase for comparative genomics [15, 16], while new functional tools such as RNAi, transgenesis, and CRISPR/Cas genome disruption and editing are revealing new information about human parasites, including new targets for intervention [17, 18]. Genome editing has also been proposed as an approach toward producing attenuated parasites for functional studies or for vaccine development [19].

## Ecology and evolution and mathematical biology

Many of our modern approaches to global deworming for soil-transmitted helminth infections and schistosomiasis are based on the modeling and population dynamics studies of Anderson and May conducted during the 1980s [20]. Mathematical biology has potential importance for all of our major neglected disease control approaches. In turn, parasites have evolved with their intermediate and definitive hosts to establish amazingly complex and intimate associations [21]. The sciences of ecology and evolution have critically important roles in promoting our understanding of the host–parasite relationship [21].

## Systems biology and immunology

Systems biology has provided a new framework for understanding complicated host–parasite interactions for malaria and other systems [22–24], while the relatively new field of systems vaccinology could transform how we select vaccine candidates and advance them through clinical development in an accelerated sequence [25]. The potential value of systems vaccinology was dramatically illustrated by a recent modification in immunizing human volunteers with the RTS,S malaria vaccine [26]. While previous immunization protocols elicited significant levels of host antibody responses, the overall level of protection was considered modest. However, through fractional dosing and alterations in immunization schedule, a significantly improved level of immunity was obtained [26]. Ideally, through systems vaccinology, such findings could have been obtained earlier in the vaccine development process. In this regard, a new Human Vaccine Project to better understand the human immune system and how it responds to vaccines has been launched [27].

The areas of basic science discussed here are by no means comprehensive but instead were selected to illustrate the enormous number of exciting findings that might lie ahead. During the 1980s, as an MD–PhD graduate student, I was privileged to work in one of the first laboratories devoted to applying the new science of molecular biology to the study of animal parasites [10]. My scientific career began as a basic scientist isolating new proteins and genes from hookworms to understand how these parasites invade human tissues. Only later did I look at how we might interfere with these molecular processes as an effort to develop new vaccines. Over time, my career transitioned from basic to translational science, ultimately folding in a program of public engagement through policy and advocacy.

Today, we are once again at a new threshold when active pursuit in the basic science areas highlighted above will provide an exciting tranche of insights for tropical disease pathogens and host–pathogen interactions. We could soon gain mission-critical information about molecular and cellular biology and how it integrates within biological systems and disease ecology. If adequately supported, these findings could jump-start a new generation of translational medicine and control tools. In so doing, we will help to ensure that a robust pipeline of neglected diseases interventions can continue. Just as the world’s poor deserve access to food, water, shelter, and essential medicines, they also deserve access to innovation [28]. We can do better to promote basic research to benefit marginalized and impoverished populations.

## Abbreviations

DALYs: disability-adjusted life years
NIAID: National Institute of Allergy and Infectious Diseases
NIH: National Institutes of Health
NTDs: neglected tropical diseases
PDPs: product development partnerships
R&D: research & development
sci-RNA-seq: single-cell combinatorial indexing RNA sequencing
